# Assessment of the Implementation of Pakistan’s National Action Plan on Antimicrobial Resistance in the Agriculture and Food Sectors

**DOI:** 10.3390/antibiotics13030206

**Published:** 2024-02-22

**Authors:** Yu Qiu, Jorge Pinto Ferreira, Riasat Wasee Ullah, Peter Flanagan, Muhammad Usman Zaheer, Muhammad Farooq Tahir, Javaria Alam, Armando E. Hoet, Junxia Song, Muhammad Akram

**Affiliations:** 1 Food and Agriculture Organization of the United Nations (FAO) Headquarters, 00153 Rome, Italy; jorge.pintoferreira@fao.org (J.P.F.); junxia.song@fao.org (J.S.); 2 Office of the Animal Husbandry Commissioner, Ministry of National Food Security and Research, Government of Pakistan, Islamabad 44000, Pakistan; riasatwasee@gmail.com (R.W.U.); ahc.cvopakistan@gmail.com (M.A.); 3 FAO Regional Office for Asia and the Pacific, Bangkok 10200, Thailand; peter.flanagan@fao.org (P.F.); and muhammad-usman.zaheer@fulbrightmail.org (M.U.Z.); 4 FAO Country Representative Office, Islamabad 44000, Pakistan; drmftahir@gmail.com (M.F.T.); javaria.alam@fao.org (J.A.); 5 FAO Reference Center on Antimicrobial Resistance, Department of Veterinary Preventive Medicine, College of Veterinary Medicine, The Ohio State University, Columbus, OH 43210, USA; hoet.1@osu.edu

**Keywords:** antimicrobial resistance, national action plan, implementation assessment, agriculture and food, Pakistan

## Abstract

The agriculture and food (agrifood) sectors play key roles in the emergence, spread, and containment of antimicrobial resistance (AMR). Pakistan’s first National Action Plan (NAP) on AMR was developed to guide One Health interventions to combat AMR through 2017–2022. To improve subsequent iterations, we assessed the implementation of Pakistan’s NAP in the agrifood sectors (NAPag) in October 2022, using the Progressive Management Pathway on AMR tool developed by the Food and Agriculture Organization of the United Nations (FAO). The assessment tool addressed four crucial focus areas of the NAPag: governance, awareness, evidence, and practices. Each focus area contains multiple topics, which involve four sequential stages of activities to progressively achieve systematic management of AMR risk in the agrifood sectors. High-level representatives of the NAPag stakeholders provided information for the assessment through pre-event documentary review and workshop discussions. The assessment results showed that Pakistan’s NAPag had an overall moderate coverage (59%) of the anticipated activities. Gaps were particularly notable in strengthening governance, good practices, and interventions in non-livestock sectors. Furthermore, only 12% of the evaluated activities were fully executed and documented, consistently remaining at the planning and piloting stages in the livestock sector across all the examined topics. Insufficient attention to non-livestock sectors, inadequate regulation and enforcement capacity, and resource constraints have hindered scalable and sustainable interventions under the current plan. This assessment provides valuable insights to strengthen the inclusiveness and contribution of the agrifood sectors in the next NAP iteration. In the short-to-medium term, strategic prioritization is necessary to optimize the use of limited resources and target the most critical gaps, such as improving awareness among key stakeholders and fortifying regulations for prudent antimicrobial use. In the long term, integration of AMR into the country’s broader health, development, and agricultural transformation agendas will be needed to generate sustainable benefits.

## 1. Introduction

Antimicrobial resistance (AMR) is a leading global health threat that can spread across humans, animals, plants, and the environment. Bacterial AMR was estimated to be directly responsible for 1.27 million human deaths worldwide in 2019, with the highest burden borne by low- and middle-income countries (LMICs), particularly in sub-Saharan Africa and South Asia [1]. The World Bank forecasts that AMR could cause low-income countries to lose more than 5% of their gross domestic product (GDP) and push up to 28 million people into poverty by 2050 [2]. Antimicrobial resistance also threatens the sustainability of animal and crop production and environmental health. According to the World Bank, the reduction in productivity arising from resistant infections and the associated international trade disruptions could decrease the annual global livestock production between 2.6% and 7.5% by 2050 [2].

Many antimicrobials are shared in agriculture and human medicine [3], and their overuse and misuse in humans and agriculture are accelerating the proliferation of AMR. Resistance can spread across sectors via multiple routes, including direct contact, food chain, and environmental pathways such as contaminated water. Scientific evidence shows antimicrobial use (AMU) in animals is associated with the occurrence of AMR in animals [4,5] and, for certain antimicrobials, in humans [6,7,8]. This underscores the need to address AMR through an integrated One Health approach.

Pakistan is a lower-middle-income country in South Asia with a population of over 240 million inhabitants, making it the fifth most populous country in the world. Agriculture plays a key role in Pakistan’s economy, employing 37% of the workforce and accounting for 23% of its GDP in 2021–2022 [9]. The livestock sector (including poultry) is by far the largest agricultural sector, accounting for 62% of the total agricultural output and 14% of the national GDP [9]. The main food animals raised in Pakistan include poultry, cattle (primarily dairy), buffalo, sheep, and goats. While ruminants are typically kept in smallholder production systems [10], the poultry industry is shifting toward modern and intensive production [11]. Aquaculture is a recent development that is typically practiced in small-scale, extensive production systems, accounting for 1% of the country’s GDP [12].

As in many other countries, AMR poses a significant challenge to public health and sustainable agricultural development in Pakistan. The country ranks third among LMICs in terms of total antimicrobial consumption by humans, which increased by 65% between 2000 and 2015 [13]. Pakistan also has one of the highest levels of AMU in food-producing animals, with a projected 44% increase from 2020 to 2030 [14]. Increasing rates of AMR, including multidrug resistance, have been reported in both human and animal health sectors [15].

Despite escalating levels of AMR in Pakistan, the Joint External Evaluation (JEE) conducted in 2016 showed that Pakistan lacked capacity to tackle AMR, and it also stressed the urgent need to prioritize AMR activities [16]. In response to the JEE recommendations and the global call to curb the increasing risk of AMR, the Pakistan Ministry of National Health Services Regulations & Coordination (MoNHSR&C), which is the ministry in charge of public health, developed the National Strategic Framework for Containment of AMR in 2016 [17] and the National Action Plan (NAP) on AMR in 2017 [18]. Like in many other countries, the NAP is based on the 2015 World Health Organization (WHO) Global Action Plan (GAP) on AMR [19]. Box 1 displays the vision, mission statement, objectives, and strategic priorities of the NAP. The NAP includes the same five objectives mentioned in the GAP, along with seven strategic priorities aligned with these objectives, and recommends 13 approaches and 25 interventions to achieve them [18]. The proposed interventions cover primary activities, anticipated outcomes, specified timeframes, evaluation indices, and allocated responsibilities across the pertinent sectors at the federal, provincial, and district levels.

While the establishment of the NAP has created momentum to accelerate AMR activities in Pakistan, assessments are needed to identify the achievements and gaps, as well as to guide future inventions and inform the next NAP update. In 2021, Saleem et al. [20] conducted a narrative review summarizing national AMR activities and challenges aligned with the five NAP objectives, drawing from a selection of published literature. However, this review does not establish a connection with the interventions outlined in Pakistan’s NAP. In addition, most publications included in the review focus predominantly on the human health sector. Consequently, the assessment has limited coverage of the agriculture and food (agrifood) sectors, notwithstanding their important role in the emergence and spread of AMR. As Pakistan’s NAP (2017–2022) approached its expiration, the Ministry of National Food Security and Research (MoNFS&R), which is the ministry in charge of agrifood, organized a national assessment in 2022, with support from the Food and Agriculture Organization of the United Nations (FAO). The objectives of the present assessment were twofold: (1) to estimate the level of inclusion of agrifood components within the NAP, and (2) to evaluate the implementation status of the NAP in the agrifood sectors, referred to as NAPag. In this paper, we present the findings of this assessment and discuss their implications.

Box 1 **The vision, mission statement, objectives, and strategic priorities of Pakistan’s National Action Plan on antimicrobial resistance (AMR) 2017–2022 [18].**

Vision:
No Pakistani should suffer from AMR in the coming years.
   
Mission statement:
To have a functional, coordinated, collaborative, and sustainable AMR containment system in place using the “One Health” approach aligned with the WHO Global Action Plan on AMR.
   
Objectives and strategic priorities:
Objective 1: Improve awareness and understanding of AMR through effective communication, education and training.
  1^st^ Strategic Priority: Development and implementation of a national awareness raising & behavioral change strategy on AMR.
Objective 2: Strengthen the knowledge and evidence base through surveillance and research.
  2^nd^ Strategic Priority: Establishment of an integrated national AMR surveillance system (human and animal usage and resistance monitoring).
Objective 3: Reduce the incidence of infection through effective sanitation, hygiene, and infection prevention measures.
  3^rd^ Strategic Priority: Improve prevention and control of infections in health care, community, animal health, food, agriculture, and the environment.
Objective 4: Optimize the use of antimicrobial medicines in human and animal health.
  4^th^ Strategic Priority: Update and enforce regulations for human and veterinary antimicrobial use.
  5^th^ Strategic Priority: Phase out the use of antimicrobials as growth promoters and provide appropriate alternatives.
Objective 5: Develop economic cases for sustainable investment based on country needs and increase investment in new vaccines, diagnostics, and other interventions.
  6^th^ Strategic Priority: Integration of AMR in all public health research agendas including research on vaccines and diagnostics.
  7^th^ Strategic Priority: Estimation of health and economic burden of AMR for decision making.

## 2. Materials and Methods

### 2.1. Assessment Tool

**Figure 1:**
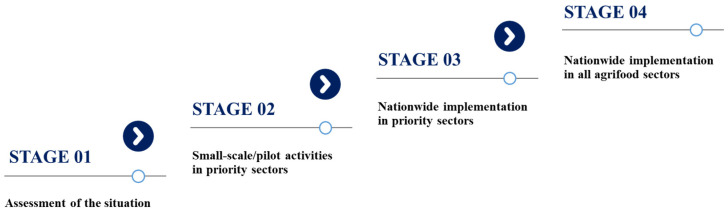
Schematic representation of the four stages and overall corresponding activities for each assessment topic. Source: FAO Progressive Management Pathway for antimicrobial resistance (AMR) [21,23].

In May 2022, the Chief Veterinary Officer (CVO) of Pakistan (M.A.) officially requested FAO’s support to conduct a national assessment of Pakistan’s NAPag implementation. In response to this request, FAO assembled a core planning team to prepare the assessment (Y.Q., J.P.F., P.F., and M.F.T.). The assessment was conducted using the Microsoft Excel-based FAO Progressive Management Pathway for AMR (FAO-PMP-AMR) tool, which has been used by more than 30 countries [21] and is described elsewhere [22,23]. The assessment tool addressed four crucial focus areas of the NAPag: governance, awareness, evidence, and practices. Each focus area contains multiple topics adapted from the 2015 WHO GAP [19] and the FAO Action Plan on AMR (2016–2020) [24] with a specific focus on the agrifood sectors (see Table 1). Each topic comprises a series of activities that are arranged into four sequential stages, offering countries a systematic framework to progressively achieve effective management of AMR risk in the agrifood sectors (see Figure 1). In brief, Stage 1 encompasses assessment of the situation, including activity planning; the subsequent stages are linked to piloting interventions in the priority sectors (Stage 2) and scaling up for national coverage in the priority sectors (Stage 3). Finally, Stage 4 involves systematic implementation of activities across all the agrifood sectors at the national level. Each activity can be assessed if it is included (or not) in the NAP and its implementation status. The tool automatically converts the results into percentages of coverage and accomplishment, which are aggregated for each focus area.

### 2.2. Assessment Workshop

Key organizations and individuals involved in the implementation of Pakistan’s NAPag were identified and invited to join the assessment as assessors. These included representatives from relevant government sectors at national and provincial levels, civil society organizations, academia and research institutions, private stakeholders, and development partners. Two weeks prior to the assessment workshop, an online training session was organized by the FAO core planning team to introduce the assessment tool to the assessors. The assessors were also informed of the importance of honest and independent feedback and the value of their input for improving the NAPag implementation. At the end of the training, the assessors were tasked with collecting the latest information related to each assessed activity under their authority, drawing from both published and unpublished documents.

The three-day assessment workshop was organized in Islamabad on 24–26 October 2022, and moderated by two facilitators from the FAO headquarters (J.P.F. and Y.Q.). The facilitators had no role in Pakistan’s NAPag implementation and no stake in the assessment outcomes. The workshop drew 46 high-level representatives from six national sectors who were directly involved in decision making on AMR-related activities at their respective public or private agencies (Table 2). However, some invitees were not able to attend the workshop, including representatives from the plant and environmental sectors, the pharmaceutical and animal feed industries, and some other private and international stakeholders.

During the assessment, the facilitators first provided an explanation for each topic under assessment and its associated activities. The participants subsequently engaged in discussions to reach a consensus on whether these activities were included in the NAP and the status of implementation, based on cross-referencing information gathered from pre-event documentary review. Half a day was allocated to each of the four focus areas to ensure in-depth discussion and broad-based deliberation on the assessment results. The assessment outcomes were shared with the workshop participants post-event for validation before receiving final endorsement from the CVO of Pakistan.

## 3. Results

### 3.1. Overview of the Sectoral Coverage and Implementation Approach in the NAP

The NAP of Pakistan focused mostly on the human and livestock health sectors. Other sectors (such as plants, aquaculture, and the environment) were rarely mentioned. There was mixed coverage of the human and livestock health sectors in the strategic, operational, and monitoring and evaluation (M&E) plans. Sectoral-specific plans for agrifood as a whole did not exist. A direct implementation approach was deployed throughout the NAP, without following sequential phases or gradual rollouts of the activities under the plan.

### 3.2. Overall Assessment Results

Overall, Pakistan’s NAP encompassed 59% of the anticipated agrifood activities as outlined in the FAO-PMP-AMR assessment tool. When analyzed across the four focus areas, coverage was higher for evidence and awareness (80% and 71%, respectively) compared with governance and practices (44% and 40%, respectively). Notably, significant gaps were identified in areas related to financial sustainability, regulatory framework, prudent AMU, and interventions in non-livestock sectors. Concerning the four advancement stages, Stage 4 activities were far less evident in the NAP compared to the preceding stages.

Implementation of the NAPag consistently occurred at low levels across all the four focus areas (ranging from 8% to 14%), with only 12% of the assessed activities completed overall (i.e., with clear evidence of execution and documentation). Most of the implemented activities were concentrated in Stages 1 and 2, encompassing baseline assessments and pilot activities in the priority sectors (i.e., poultry and dairy).

### 3.3. Subject-Specific Implementation Status

The implementation status of Pakistan’s NAPag across the assessed topics of the four focus areas is summarized under the following nine subjects.

#### 3.3.1. Governance: Multisectoral Coordination

To coordinate the NAP implementation, Pakistan established an AMR multisectoral steering committee in April 2018, comprised of representatives from human health, agriculture and food safety, and environmental sectors. The committee meets once a year to provide high-level, strategic direction on AMR activities. The National Institute of Health (NIH) under the MoNHSR&C acts as the secretariat and the national coordination center overseeing the NAP implementation. Some provinces had established their own AMR multisectoral coordination units. Specific AMR coordination mechanisms across the livestock, aquaculture, and plant sectors within the agrifood systems did not exist. Within the livestock sector, the CVO’s office coordinates AMR activities nationwide, in close collaboration with AMR focal points at the provincial livestock departments. Nevertheless, as noted by the assessors, constraints in human and financial resources had impeded the efficient functioning of these coordination mechanisms and the establishment of necessary technical working groups, resulting in a gap between discussions and tangible actions.

#### 3.3.2. Governance: Sustainability

The NAP was not linked to a budget plan or any resource mobilization strategies. The projected expenditure for implementing the NAP over five years amounted to PKR 1726.90 million (~8.68 million British pound sterling). However, in 2019, the government approved funding of only PKR 361.96 million (~1.82 million British pound sterling), with the majority of the budget allocated to the MoNHSR&C [25]. Aside from enhancing existing facilities and infrastructure, the MoNFS&R had minimal domestic resources to support the NAPag implementation, which ended up being largely financed by international donors such as the Fleming Fund, WHO, and United States Agency for International Development (USAID) [25].

Pakistan’s legislation, regulations, and policies related to AMR in the agrifood systems have been documented in the FAO AMR-LEX database [26]. They were found to be either outdated, insufficient, or inadequately implemented. In addition, they had not undergone reviews and updates in line with the standards set by the World Organisation for Animal Health (WOAH) and Codex Alimentarius, as specified in the NAP. Moreover, the regulatory authorities and MoNFS&R displayed a general lack of initiative in promoting awareness and enhancing compliance among the relevant agricultural stakeholders. While the federal government holds the responsibility of establishing the national regulatory framework for AMR, provincial governments operate autonomously and have the authority to establish their own rules. However, a national system to evaluate the alignment of regulations between the national and provincial levels or to oversee their enforcement did not exist. Despite the inclusion of an M&E plan in the NAP, continuous monitoring and periodic evaluations of the implementation did not exist in the agrifood sectors. Consequently, there was no feedback mechanism to identify gaps in implementation and to make adjustments based on the lessons learned.

#### 3.3.3. Awareness: Awareness Promotion

Baseline studies on knowledge, attitudes, and practices (KAP) conducted in 2020–2021 showed that veterinary practitioners, as well as poultry and dairy farmers in Pakistan, commonly lacked adequate training, exhibited notable knowledge gaps, and engaged in improper practices regarding AMR and AMU [27,28,29]. For example, over 70% of farmers purchased antibiotics without consulting a veterinarian [27], and more than 60% of veterinarians had never attended any training or awareness seminars related to AMR [29]. There was a shortage of information on knowledge gaps and behavioral drivers for other key agrifood stakeholders, as well as the public’s perception of food safety and the health consequences of AMR.

The NAP emphasized the need to promote awareness and instigate behavioral change among various stakeholder groups to tackle AMR. While some provinces, such as Punjab and Khyber Pakhtunkhwa, had mapped and analyzed AMR-related agrifood stakeholders, this initiative had not been expanded to encompass the entire country. The CVO’s office, provincial livestock departments, and veterinary educational institutions organize awareness-raising activities on the AMR risk, prudent and responsible AMU, and infection prevention and control (IPC) for veterinary students, practitioners, and livestock farmers. During the World Antimicrobial Awareness Week, AMR awareness is promoted intensively through seminars, competitions, and mass media [25,30,31]. However, these activities concentrated primarily in urban areas, with limited outreach to rural regions. Notably, the NAP lacked provisions for periodic assessments to measure their impact on knowledge transfer and behavioral change.

#### 3.3.4. Awareness: Education and Training

In line with the NAP, the Pakistan Veterinary Medical Council convened in 2020–2021 to review and update the core curricula for veterinary undergraduate education and continuing development programs, focusing on integrating contents related to AMR and prudent AMU. However, this review had not been completed by the time of the assessment. While provincial livestock departments regularly train farmers on good animal husbandry practices and IPC, the training did not incorporate specific topics related to AMR and judicious AMU.

#### 3.3.5. Evidence: AMU Monitoring

The NAP emphasized regular monitoring of the sale and use of antimicrobials at all levels and in all sectors, strengthening record-keeping mechanisms, and the compilation of national sale and use records. The animal health sector had mapped the flow of antimicrobial production, importation, and distribution at the national level. However, data regarding the overall sale of antimicrobials were not available at the national or provincial level; furthermore, national systems to record veterinary prescription data or collect AMU data at the farm level did not exist [32], aside from limited project-based farm surveys. According to the assessors, the Pakistan government had insufficient capacity to inspect retailers and feed mills for veterinary drugs including antimicrobials, or to monitor drug residues in food products of animal origin. Information on AMU was not available in other agrifood sectors.

Since 2016, the CVO’s office has reported baseline information on AMU in animals to WOAH annually. In 2021, Pakistan submitted some quantitative AMU data through the WOAH Reporting Option 1 for the first time [33,34]. The total volume of AMU in animals was estimated from import data and sample surveys. This information was categorized by antimicrobial class and type of use (veterinary medical use or growth promotion) without differentiating animal species, production context, and routes of administration [34]. Annual reports on the AMU trend in animals did not exist.

#### 3.3.6. Evidence: AMR Surveillance

The NAP aimed to establish reference laboratories and surveillance networks across all sectors, as well as to develop surveillance plans, protocols, and quality assurance systems. Noteworthy progress has been made in the animal health sector. The national laboratory networking group for animal health, composed of representatives from various agencies, was established in 2020 to provide guidance on laboratory activities related to animal diseases and AMR. Two national reference laboratories (NRLs) on AMR, namely the National Veterinary Laboratory and the National Reference Laboratory for Poultry Diseases, had been designated. Additionally, many veterinary laboratories in the provincial and district livestock departments, academic institutions, and private sectors have capability to perform microbiological and susceptibility testing of common pathogens. National AMR surveillance strategies for healthy food animals, diseased food animals, the food animal environment, and aquaculture were developed during 2020–2022. Some provinces had subsequently formulated their own provincial plans for AMR surveillance in animals aligned with the national strategies. National standard operating procedures (SOPs) for sample collection and shipment, bacteria isolation and characterization, laboratory biosecurity, antimicrobial susceptibility testing (AST), and data recording were established by the NRLs during 2020–2022. While the two NRLs had received International Organization for Standardization (ISO) 17025 accreditation, not all peripheral laboratories at the district and provincial levels possessed the capacity and capability to generate, record, and report resistance data in accordance with the national SOPs. To address this deficiency, capacity-building programs had been conducted [30,35,36]. Furthermore, nine provincial laboratories had been enrolled in the laboratory quality management system, which includes external quality assessment.

While the NAP aimed to establish an integrated national AMR/AMU surveillance system spanning both human and animal health, a national surveillance system to routinely monitor AMR in animals did not exist. Surveillance of AMR in animals was mainly passive and localized. According to the assessors, AST was carried out primarily in bacterial pathogens from food-producing animals to guide clinical treatment (often without strict adherence to SOPs) and, to a lesser extent, for research. Despite the patchy surveillance, alarming results have been reported. For example, a local survey showed over 50% prevalence of multidrug resistance in *Campylobacter jejuni* from slaughtered healthy broilers and cattle fecal samples [37]. Also, 54% prevalence of methicillin-resistant *Staphylococcus aureus* (MRSA) was identified in milk samples from dairy cattle with subclinical mastitis in the Pothohar region of Punjab [38]. Since July 2020, the government has initiated a pilot, active AMR surveillance in commensal and foodborne zoonotic bacteria from apparently healthy broilers, cattle, and buffaloes at slaughterhouses [30]. The preliminary results showed high levels of resistance to some WHO critically important antimicrobials (CIAs) such as cefotaxime, nalidixic acid, and streptomycin among *Escherichia coli* and *Salmonella* spp. isolates [35,36]. Studies of retail food commodities (e.g., meat, dairy products, and fruits) have also shown high rates of resistant bacteria (e.g., *Campylobacter*, *E. coli*, *Shigella*, and *Salmonella*) to commonly used antibiotics [39,40,41,42,43]. Information on AMR prevalence in the plant, aquaculture, and environmental sectors is relatively limited. Still, sporadic studies reported high rates of multidrug-resistant bacteria in fish [44,45,46], and consistently higher resistance rates to commonly used antibiotics in soil *Pseudomonas aeruginosa* isolates within 25 m of poultry farms [47]. Notably, AMR surveillance activities in different sectors were reported by the assessors as mostly operated in silos with limited cross-sectoral collaboration. The WHO-funded extended-spectrum beta-lactamase (ESBL)-producing *E. coli* Tricycle project [48] represents a pilot of integrated surveillance encompassing the human, animal, and environmental sectors. The preliminary, unpublished results from this project indicated high rates of genotypic ESBL-producing *E. coli* from all the three sectors in 2018.

**Figure 2:**
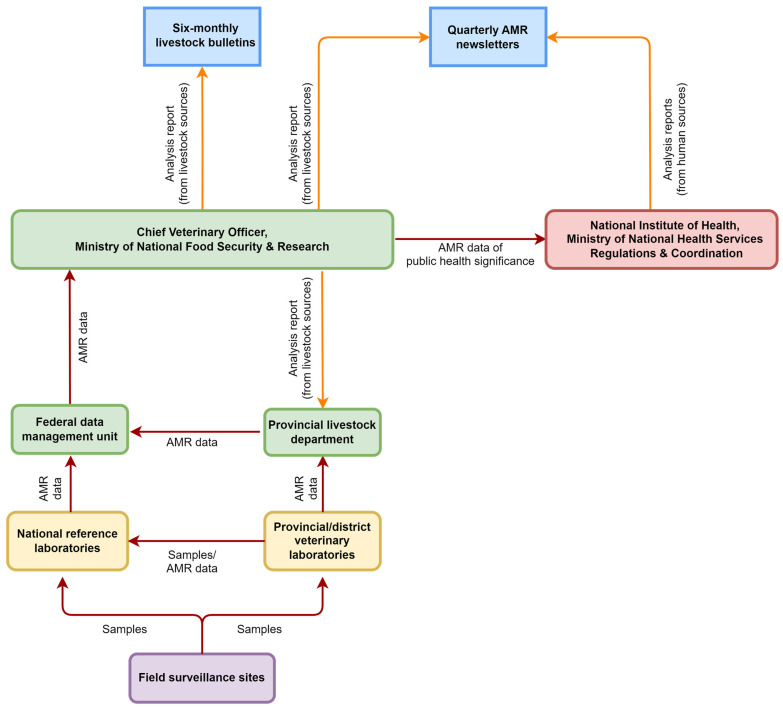
Antimicrobial resistance (AMR) reporting framework in the Pakistan livestock sector.

The NAP aimed to develop mechanisms for data reporting in each sector and create a common dashboard for data sharing among public and private stakeholders at all levels. The AMR data reporting structure was subsequently established in the livestock sector in 2021, as illustrated in Figure 2. However, the assessors noted that this framework primarily captured active surveillance data from government-coordinated programs, while data from passive surveillance or laboratories in the private sectors and academic institutions were not incorporated. This limitation has contributed to the absence of annual reports detailing the prevalence and trends of AMR in the country. Consequently, most of the available AMR epidemiological data were derived from isolated studies instead of integrated central analysis.

#### 3.3.7. Evidence: AMR Research and Economic Studies

The NAP underscored innovating new vaccines, diagnostics, and alternatives to antimicrobials, as well as conducting social science studies and economic research. However, a national AMR priority research agenda outlined in the NAP had not been developed by the time of the assessment. Furthermore, the assessors noted that the government’s support for AMR research was limited, with most initiatives spearheaded by veterinary and medical academic institutions. Research on the economic impacts of AMR and incentives for prudent AMU in agriculture was notably absent. 

#### 3.3.8. Practices: Good Agricultural Practices

The NAP emphasized the enhancement of IPC policies, guidelines, and practices across all sectors, which includes promoting disease prevention measures like sanitation practices and animal vaccination. The federal and provincial veterinary authorities actively advocate for good husbandry practices, farm biosecurity, and vaccination, through various means such as training courses, awareness seminars, extension services, and disease control programs. Also, the government periodically issues relevant guidelines, adjusting to the dynamic disease situation, risk factors, and specific needs. Pakistan has capacity in vaccine production and the implementation of vaccination programs for common livestock diseases such as foot and mouth disease, hemorrhagic septicemia, anthrax, brucellosis, avian influenza, and Newcastle disease (https://vri.punjab.gov.pk/system/files/VRI%20Annual%20Report%202020-2021.pdf; accessed on 10 December 2023). Nonetheless, challenges such as resource constraints, weak infrastructure, and knowledge gaps among livestock farmers have hindered the realization of its full potential, resulting in insufficient vaccination coverage and recurrent disease outbreaks [15,49].

The NAP incorporated the adoption of waste management practices consistent with the Pakistan Environmental Protection Act 1997. Certain provinces had developed their own regulations, such as the Punjab Poultry Production Act 2016 that regulates waste disposal from poultry farms. However, these regulations were noted to be at the early stages of implementation. It has also been reported as a common practice to use untreated cattle and poultry manure as nutrient input for fish production and as crop fertilizer [28,50], which can potentially facilitate AMR spread.

#### 3.3.9. Practices: Prudent AMU

The NAP underlined the need to update and enforce regulations on the quality control, sale, prescription, and use of human and veterinary antimicrobials. The Drugs Act 1976 and the Drug Regulatory Authority of Pakistan (DRAP) Act 2012 set the legal requirements for the manufacture, import, export, quality control, marketing authorization, and sale of antimicrobials, but wide gaps have been reported in the implementation [17,32]. Surveillance aiming at preventing the circulation of substandard or falsified antimicrobials in the market is inadequate [51]. While some antibiotics are legally dispensed only with a valid prescription, over-the-counter (OTC) sale remains prevalent [52]. A general lack of regulation and supervision of antimicrobial prescription and use in animals has been a concern [15,17,32]. Veterinary prescriptions are typically issued without microbiological and susceptibility testing and tend to use broad-spectrum antibiotics [15]. Previous surveys showed that more than 50% of interviewed veterinarians and farmers prescribed or used antibiotics for inappropriate conditions such as viral diseases [28,29]. Studies in Punjab, the largest and most populous province of Pakistan, showed a significantly higher relative AMU (measured as the quantity of antimicrobial active ingredients adjusted for animal biomass) in commercial broilers and dairy cattle than the global averages [10,53,54]. In 2022, Pakistan embarked on a national consultative process for the development of antimicrobial prescribing guidelines for livestock, although these guidelines had not been finalized by the time of the assessment. The growing aquaculture industry in Pakistan also fuels an increased reliance on antimicrobials to prevent and treat diseases [15,46]. National guidelines for AMU in this sector had not been developed at the time of the assessment.

In Pakistan, the major share of AMU in animals is for prophylaxis (i.e., disease prevention) and growth promotion, routinely administered at subtherapeutic levels in feed or water for extended duration [32]. The relevant regulatory framework did not exist except for Punjab province, where a list of specific antimicrobial growth promoters (AGPs) permitted as feed additives had been issued (Table 3). At the time of the assessment, Pakistan did not have its own nationally defined medically/critically important antimicrobials for human medicine or veterinary medicine. Also, the NAP did not encompass the regulation of the use of WHO CIAs in non-human sectors, despite their widespread and indiscriminate use in animals [29,55]. Surveys conducted at the farm level for broilers [54] and dairy cattle [10] production showed that WHO CIAs constituted 60% and 42% of AMU, respectively.

Although antimicrobial stewardship was emphasized by the NAP for both human and animal health sectors, it was lacking in most animal health establishments, coupled with a low level of awareness and application among veterinary practitioners [29]. Moreover, inadequate awareness and regulation regarding Maximum Residue Limits (MRLs) in food have been reported [32], coupled with inadequate compliance with antibiotic withdrawal periods [28]. This lack of adherence to withdrawal periods and the excessive use of antibiotics have been reflected in studies that show residues of commonly used antibiotics surpassing MRLs in various food commodities across the country [56,57,58]. No initiatives were in operation to benchmark veterinary professionals and farmers on AMU for peer comparison as described elsewhere [59], leading to a lack of targeted interventions for high users. While the NAP did not explicitly address the disposal of leftover or expired antimicrobials, malpractice in this regard is widespread [29].

## 4. Discussion

The present assessment provides detailed insights into the integration of agrifood components in Pakistan’s NAP as well as the progress and gaps after 5 years’ NAPag implementation. As a strategic document, the NAP is central to the fight against AMR, representing commitments from decision-makers that AMR is receiving an appropriate level of priority and support. This, in turn, should facilitate the mobilization of resources and the impactful implementation of corresponding actions. However, the greatest challenge is often not writing a NAP, but translating it into sustained actions on the ground, according to the Interagency Coordination Group on AMR [60]. Pakistan’s NAP was formulated in accordance with the guidelines from the WHO GAP (2015) [19] and AMR Manual for developing NAPs (2016) [61]. It covered a moderate proportion (59%) of the anticipated agrifood activities specified in the FAO-PMP-AMR assessment tool. Although Pakistan has made some progress, the overall NAPag implementation level was low (12%) and consistently remained at the early stages (i.e., assessment and pilot activities) across all the topics. This aligns with Pakistan’s self-reported NAP implementation to the Tracking AMR Country Self-Assessment Survey (TrACSS), in which the national capacity and progress in the animal health and other agrifood sectors were mostly said to be limited and below the global averages [62]. In the present assessment, significant gaps in Pakistan’s NAPag implementation were identified across the focus areas of governance, awareness, evidence, and practices. These findings concur with previous reports [15,32], but offer more up-to-date and detailed information, providing decision-makers with more precise insights for devising targeted interventions. The impartiality of the participant responses and unbiased information within this assessment were enhanced through third-party facilitators from the FAO headquarters, a diverse representation of participants from various backgrounds and sectors, cross-referencing information, and post-event validation. Moreover, the present assessment has unique features to strengthen the ownership of results by the participating sectors, and to foster consensus building and collaboration between different agencies.

The WHO GAP advocates for a multisectoral One Health approach to combat AMR [19]. The NAP of Pakistan was developed through a cross-sectoral approach. However, some sectors, such as the ministries of finance and education, were less involved, resulting in their suboptimal engagement. Our assessment showed that Pakistan’s NAP mostly focused on human and livestock health, paying insufficient attention to plants, aquaculture, companion animals, food and feed production, and the environment. Similar gaps have been reported by many other countries [63,64]. The insufficient attention paid to these sectors can be attributed, at least partially, to a lack of evidence and awareness regarding their significance in AMR risk management. The absence of these sectors from the present assessment process may reflect their weak perception of the relevance and ownership of the NAP, which prevents a more comprehensive evaluation of the NAPag implementation. Additionally, Pakistan faces challenges in implementing the decisions of the AMR multisectoral steering committee, due to insufficient dedicated human and financial resources, under-invested infrastructure, weak stakeholder engagement, outdated regulatory framework and inadequate enforcement capacities, which are common challenges in many LMICs [65,66]. Another significant challenge lies in ensuring the sustainability of the NAPag implementation. The AMR activities in Pakistan so far have depended predominantly on external funding such as the Fleming Fund. Addressing how to maintain progress after the conclusion of external projects underscores the importance of enhanced government commitment, internal financing, and creative planning. Furthermore, it is imperative to update and enforce AMR-related laws and regulations in Pakistan, aligning them with sustainability goals related to IPC practices, prudent AMU, and AMR and AMU surveillance in the agrifood systems.

Effective advocacy, communication, education, and training are vital for improving awareness of AMR and facilitating behavioral change. Similar to other South Asian countries such as Bhutan [67], Bangladesh [68], and Nepal [69], Pakistan has identified substantial gaps in AMR/AMU-related knowledge and practices among veterinarians and livestock farmers [27,28,29]. These studies provide essential baseline data and offer valuable insights for designing interventions and policies to address the overuse and misuse of antimicrobials in livestock production. Like many other LMICs [70,71,72], resource and infrastructure limitations impede the implementation of comprehensive training and awareness-raising programs among a broader range of stakeholders in Pakistan, particularly in rural areas. To address this challenge, it is necessary to systematically integrate AMR into the local and national education and training programs across all the disciplines of agrifood production. Accessible online training and tailored educational approaches can be implemented to enhance cost-effectiveness and reach a wider audience.

Pakistan has not established a national system to routinely monitor AMU in animals, including the collection of AMU data at the farm or animal species level, which is a common gap in LMICs [73]. This absence prevents precise AMU trend analysis, comparison of AMU data across farms and sectors, and study of the relationship between AMU and AMR, ultimately limiting the government’s effort to reduce and rationalize AMU. Many countries collect and monitor data on the total sales of antimicrobials in animals, which is useful for analyzing the overall trend of AMU [74,75]. However, sales data alone do not contain end-use information on the quantities and drivers of AMU in different species and production types. The veterinary electronic prescription system has been utilized in some countries such as Denmark, Sweden, and Italy [75,76]. It has the advantages of allowing the collection of AMU data with sufficient details, improving the quality of antimicrobial prescribing, and facilitating targeted interventions to optimize AMU. However, this approach alone is not enough in countries including Pakistan, where farmers often do not consult veterinarians to use antimicrobials [27,28,77,78]. Many countries in Europe and North America have established systems to monitor farm-level AMU in all or certain food-producing animal species (mostly pigs and broilers) through one or various data sources (e.g., treatment/prescription records, sales/purchase data, etc.), and their experience has been summarized elsewhere [79,80]. The FAO and WOAH have recently developed a joint technical guideline on the collection, management, analysis, and communication of farm-level AMU data [81], which will support the generation of standardized and comparable AMU data. In Pakistan, the government sector faces significant resource constraints while the poultry industry is powerful and resourceful. Hence, it may be practical to start establishing AMU surveillance in the poultry sector through a partial coverage system. This system can be fully or partially funded by private organizations and target farms that adhere to the respective quality assurance scheme, as in some European countries [80].

Establishing a robust AMR surveillance system is critical for assessing the burden of AMR, informing strategies to contain AMR, and evaluating the effectiveness of interventions. The JEE report (2016) highlights the urgent need for Pakistan to bolster the diagnostic laboratory infrastructure in both human and animal health sectors, and to standardize the AST procedures and interpretation [16]. In response to the JEE recommendations, the Pakistan AMR Surveillance System (PASS) has been developed to collect AMR data from priority human pathogens [82]. Pakistan also reports AMR surveillance data from humans to the WHO Global Antimicrobial Resistance and Use Surveillance System (GLASS) [83]. However, similar national AMR surveillance systems have not been developed for the animal health and agriculture sectors, where surveillance activities remain scattered and fragmented. The lack of standardization and integration represents one of the common challenges in AMR surveillance [84,85,86,87]. The Pakistan government has endeavored to support the standardization of AMR data collection in public sector veterinary laboratories through approaches such as SOP development, training programs, and quality assurance systems. Nevertheless, like many other LMICs, Pakistan’s veterinary laboratory systems face infrastructure weaknesses, supply shortages, insufficient trained personnel, limited funding support, and poor data management [69,84,85]. These challenges constrain their ability to generate good-quality AMR data, especially in the peripheral laboratories. Additional initiatives are required to invest in laboratory resources and foster networking among laboratories to improve their contribution to national AMR surveillance. Moreover, a centralized database is urgently needed to store and manage AMR data from various administrative levels and surveillance programs, improve the harmonization and use of surveillance data, and enable data linkages and comparison between different sectors. As a constructive step, Pakistan has recently joined the International FAO Antimicrobial Resistance Monitoring (InFARM) System [88], which aims to guide and support countries to build their own national AMR surveillance databases, strengthen surveillance capacities, and improve data management in the agrifood sectors.

Disease prevention measures, including optimized management practices, nutrition, veterinary services, biosecurity, and vaccination, can effectively reduce the need for antimicrobials in food-producing animals from both high- and lower-income countries [89,90,91]. These measures also offer additional benefits by improving animal welfare and productivity, lowering costs, and simultaneously minimizing the transmission of AMR to humans and the environment. Despite the existence of a diverse array of tools and practices for disease prevention, continued efforts are required in Pakistan to enhance their accessibility and encourage farmers to adopt them. As the leader in the global effort to tackle AMR in the agrifood systems, FAO recently formulated a 10-year global initiative entitled “Reduce the Need for Antimicrobials on Farms for Sustainable Agrifood System Transformation (RENOFARM)” [92]. Under this initiative, FAO will expand its support to countries by developing and implementing customized approaches on farms to achieve healthier production while reducing reliance on antimicrobials.

A global downward trend of AMU in animals over recent years has been reported by WOAH, as a result of collective interventions including the prohibition of AGPs [34]. Many countries, such as the European Union members, China, Thailand, Vietnam, and Israel, have banned AGPs completely [93,94,95]. Still, 41 countries reported AGP use to WOAH in 2021 [34]. In Pakistan, AGPs constitute an important proportion of AMU in animals [32]. Punjab province has made a commendable move by regulating AGPs in animal feed and prohibiting the inclusion of WHO CIAs (Table 3). Further efforts are needed to extend this regulation and practice to the national level and to progressively achieve a total ban on AGPs.

The present assessment highlights significant gaps in Pakistan to restrict indiscriminate and excessive AMU in livestock. Urgent attention is required to address critical issues such as low levels of AMR awareness and risk perception among farmers and veterinarians, inadequate policies governing AMU, and unregulated OTC sale of antimicrobials. Meanwhile, interventions should consider the local context to gain buy-in from stakeholders and ensure continued accessibility to these essential drugs, particularly in areas with insufficient animal and agricultural health services. Measures are also needed to guide the prudent use of antimicrobials when treatment is required, such as improved access to reliable microbiological and susceptibility testing, implementing antimicrobial prescription guidelines, and establishing antimicrobial stewardship programs. Integration of the environmental sector into IPC and prudent AMU programs is also critical. Proper disposal of antimicrobial leftovers and farm effluent is necessary to minimize the risk of accumulation of antimicrobial residues in the environment and the establishment of environmental reservoirs for resistant microorganisms and resistance genes [96,97].

Our assessment shows that significantly fewer activities were executed under the fifth objective of Pakistan’s NAP in comparison with the preceding objectives. This discrepancy underscores a potential lag or reduced emphasis on the corresponding sixth and seventh strategic priorities, which focus on strengthening research and economic/impact studies for sustainable investment to counter AMR (Box 1). Interventions are needed to address this disparity for a more balanced NAPag implementation, as each strategic priority plays a crucial role in achieving the overarching goal of mitigating AMR risks. In addition, AMR activities need to expand beyond livestock to encompass other agrifood sectors. These activities should likewise involve strengthening governance, awareness, and robust surveillance, along with promoting good practices and responsible AMU. Fostering research and innovation in alternative disease management measures, such as integrated pest management (IPM) and biological control in crop production, can support AMR risk mitigation in a more economic and sustainable way [98]. Effective partnership among government agencies, private stakeholders, and research institutions is essential for developing and implementing comprehensive strategies to address AMR across diverse components in the agrifood systems.

## 5. Limitations

Representatives from some important national sectors (such as plant and environmental health, pharmaceutical and animal feed industries) and international stakeholders (such as WHO, WOAH, and the United Nations Environment Programme) were missing from the assessment process. This affects a more well-rounded perspective on the NAPag implementation and the overall comprehensiveness of this assessment.

## 6. Conclusions

This assessment shows that although Pakistan has made some progress in the NAPag implementation, there are significant challenges and gaps that need to be addressed to achieve scalable and sustainable impacts. The insights gained from this assessment are instrumental for guiding the next NAPag iteration in Pakistan, building on the initial achievements to design and implement more comprehensive programs. We believe that the findings of this assessment in the context of Pakistan, along with the associated implications discussed, have broad relevance for other LMICs facing similar challenges and gaps. To advance the NAPag implementation in the short-to-medium term, strategic planning is essential to maximize the efficiency of limited resources. This involves prioritizing efforts toward the most critical areas, such as improving awareness among key stakeholders and fortifying regulations for prudent AMU. In the long term, systematic integration of AMR into the country’s broader health, development, and agricultural transformation agendas will be needed to generate sustainable benefits, particularly in resource-limited settings like Pakistan.

## Figures and Tables

**Table 1:** Summary of the assessment topics. Source: FAO Progressive Management Pathway for antimicrobial resistance (AMR) [21,23].

Focus Area	Subject	Topic
Governance	Multisectoral coordination	Establishment of the multisectoral coordination mechanism
		Functioning of the multisectoral coordination mechanism
	Sustainability	Financial sustainability
		Regulatory framework
		Monitoring and evaluation
Awareness	Awareness promotion	Stakeholder mapping
		Knowledge, attitudes, and practices assessment
		Awareness-raising activities
	Education and training	Education and training of agricultural health workers
		Training of farmers
Evidence	Antimicrobial use (AMU) monitoring	Antimicrobial supply chain mapping
		AMU data collection
		AMU data reporting
	AMR surveillance	Establishment of AMR surveillance network and strategies
		Laboratory capacity building
		AMR surveillance activities
		AMR data reporting
	AMR research and economic studies	Development of national AMR priority research agenda
		AMR and AMU research activities and economic studies
Practices	Good agricultural practices	Health promotion and disease prevention measures
		Farm waste management
	Prudent AMU	Authorization, distribution, sale, and prescription of antimicrobials
		Regulation to restrict irrational use of antimicrobials
		AMU benchmarking system
		Disposal of leftover or expired antimicrobials

**Table 2:** Agencies participating in the workshop to assess the implementation of Pakistan’s National Action Plan on antimicrobial resistance in the agriculture and food sectors, 2022.

Sector	Agency	No. of Participants
**National:**		
**Animal health and food safety**	Ministry of National Food Security and Research (MoNFS&R)	3
	National Veterinary Laboratory	5
	Provincial Livestock and Dairy Development Departments	16
**Drug administration**	Drug Regulatory Authority of Pakistan (DRAP)	1
**Public health**	National Institute of Health (NIH), Ministry of National Health Services Regulations & Coordination (MoNHSR&C)	2
**Academia and research**	Poultry Research Institute, Punjab	3
	National Agricultural Research Centre	4
	University of Agriculture Faisalabad	2
	Government College University Lahore	1
**Private industry**	Nestlé Pakistan Ltd, Lahore, Pakistan	2
	Alltech Pakistan, Islamabad, Pakistan	2
**Non-profit agencies**	Civil Society Organizations	5
**Subtotal**		46
**International:**		
**Development partners**	Fleming Fund Country Project	2
	Food and Agriculture Organization of the United Nations	8
**Subtotal**		10
**Total**		56

**Table 3:** Approved antimicrobial growth promoters (AGPs) for use as feed additives in Punjab province, Pakistan.

Active Ingredient	Categorization of Importance for Human Medicine by the World Health Organization	Maximum Concentration in Feed
Lincomycin	Highly important	4.4 PPM
Enramycin	Not medically important	10 PPM
Zinc Bacitracin	Not medically important	50 PPM
Bacitracin Methylene Disalicylate	Important	50 PPM
Virginiamycin	Highly important	20 PPM
Avilamycin	Not medically important	30 PPM
Flavomycine (Bambercin)	Not medically important	12 PPM

## Data Availability

The original data generated from this assessment are not publicly available due to privacy restrictions.
